# Medication‐related osteonecrosis of the jaw and successful implant treatment in a patient on high‐dose antiresorptive medication: A case report

**DOI:** 10.1002/cre2.620

**Published:** 2022-07-27

**Authors:** Camilla Ottesen, Sanne W. M. Andersen, Simon S. Jensen, Thomas Kofod, Klaus Gotfredsen

**Affiliations:** ^1^ School of Dentistry, Section for Oral Health, Society and Technology, Institute of Odontology, Research Area Oral Rehabilitation, Faculty of Health and Medical Sciences University of Copenhagen Copenhagen Denmark; ^2^ Department of Oral & Maxillofacial Surgery Copenhagen University Hospital Copenhagen Denmark; ^3^ Section for Oral Biology and Immunopathology, Institute of Odontology, Research Area Oral Surgery, Faculty of Health and Medical Sciences University of Copenhagen Copenhagen Denmark

**Keywords:** denosumab, dental implants MRONJ, osteonecrosis of the jaw

## Abstract

**Objectives:**

Oral rehabilitation can be a challenge in patients on high‐dose antiresorptive medication (HDAR), especially if the alveolar anatomy has changed due to previous medication‐related osteonecrosis of the jaw (MRONJ) resection. In healthy patients, dental implant treatment has found wide acceptance in prosthetic rehabilitation as it increases the patient's oral health‐related quality of life. However, it is considered contraindicated in patients on HDAR due to the risk of MRONJ, although a recent feasibility study indicates that implant treatment may indeed be an option in these patients. The aim of the present case report is to illustrate the risk of MRONJ in a patient with cancer on HDAR and to discuss the reasons behind the outcomes of the implant treatment.

**Materials and Methods:**

A patient with prostate cancer with bone metastases on high‐dose denosumab therapy with previous MRONJ had four implants inserted bilaterally in the maxilla (14, 13, 23, 24). Two identical implant‐supported screw‐retained cantilever bridges were fabricated. The patient was followed for more than 1 year.

**Results and Conclusion:**

Peri‐implantitis, and/or MRONJ, was diagnosed around two of the implants (23, 24), probably induced by crestal bone trauma from a healing abutment and/or a misfitting prosthetic reconstruction. A peri‐implantitis operation was performed, but without the desired response, and the two implants (23, 24) were later removed in an MRONJ resection. The implants on the other side of the maxilla (14, 13) remained without complications. Dental implant treatment is feasible in patients on HDAR, but comorbidities (e.g., diabetes mellitus) and polypharmacy (e.g., chemotherapy and steroids) may add to the risk of implant failure. Minimal trauma surgery and prosthodontics are crucial to increase the chance of successful healing in an HDAR patient.

## 1 INTRODUCTION

Dental implant treatment in patients on high‐dose antiresorptive medication (HDAR) has been considered contraindicated due to the well‐known risk of medication‐related osteonecrosis of the jaw (MRONJ) (Stavropoulos et al., 2018). However, a recent feasibility study indicates that implant treatment may be an option in patients on HDAR (Andersen et al., 2022). In healthy patients, bone augmentation and dental implants would often be the treatment of choice to improve oral function. In patients on HDAR, the choice of treatment is frequently removable dental prostheses (RDPs) or no replacement of the lost tooth/teeth—treatment options that potentially decrease the oral health‐related quality of life (OHRQoL) of patients. Several studies have demonstrated that implant‐supported prosthodontics are related to an increased OHRQoL compared with no replacement or RDPs (Debaz et al., 2015; Øzhayat & Gotfredsen, 2020).

Removal of teeth and/or resection of necrotic bone often leaves the MRONJ patients partially edentulous and with local alveolar defects. The changed alveolar anatomy and the position of the remaining teeth often make it difficult to treat these patients with fixed dental prostheses (FDPs). RDPs may therefore be the first choice of treatment, however, they increase the patients' risk of MRONJ, especially if the fit or stability of the RDP is insufficient (Walter et al., 2016). Theoretically, dental implants can stabilize and support FDPs as well as RDPs and thereby distribute the pressure to the tissues in a favorable way, decreasing the risk of MRONJ and increasing the OHRQoL.

A prospective study was designed to evaluate the outcome of implant treatment for cancer patients on HDAR. To be included in that study, health‐related inclusion and exclusion criteria were defined (Andersen et al., 2022). One patient who did not fulfill the inclusion criteria was initially included in the study but was excluded again when the medication and comorbidities were double‐checked.

This case report presents the above patient, who went through a successful implant treatment on the right side of the maxilla whilst developing MRONJ related to the dental implants on the left side. The aims of the case report are to illustrate the risk of MRONJ in a patient with cancer on HDAR and to discuss potential reasons behind the development.

## 2 CASE PRESENTATION

### 2.1 Preimplant treatment

A 71‐year‐old man diagnosed with prostate cancer with bone metastases was in December 2016 referred from the Department of Oncology to the Department of Oral and Maxillofacial Surgery, Copenhagen University Hospital due to MRONJ in the mandible. The patient was partially edentulous and had RDPs in the upper and lower jaws. He had received HDAR, densoumab (Xgeva® 120 mg/month) for 11 months at referral. Denosumab was discontinued at the time of referral. The patient was diagnosed with nonexposed MRONJ bilaterally in the mandible due to trauma from an insufficient RDP. The clinical examination also revealed a fractured root 23, which was planned for surgical removal, at the same session as treating MRONJ in the lower jaw. He was treated surgically, healed uneventfully in all regions, and was considered cured in March 2017. The patient initiated prosthetic treatment with new RDPs at the Department of Oral Rehabilitation, University of Copenhagen. Denosumab treatment was resumed after 6 months postoperative follow‐up.

At the 1‐year follow‐up examination, the patient expressed dissatisfaction with poor retention of the upper RDP. Therefore, he was invited to participate in a research study aiming to investigate dental implant treatment in patients on HDAR. The study was designed as a prospective cohort study and approved by the Danish National Committee on Health Research Ethics (No. H‐17025531) and by the Danish Data Protection Agency (No. 2012‐41‐0045). The study followed the Declaration of Helsinki (2013) and is registered in clinicaltrials.gov (NO. NCT04741906). The patient gave his informed consent to participate in the cohort study. Unfortunately, the patient at the time of inclusion did not inform that he was diagnosed with diabetes. Diabetes was one of the exclusion criteria of the study. Therefore, the patient was excluded from the study, but at that time the patient had already had the dental implant surgery. Thus, the patient was followed thoroughly according to the study protocol, as if he was included in the study.

### 2.2 Implant insertion

**Figure 1:**
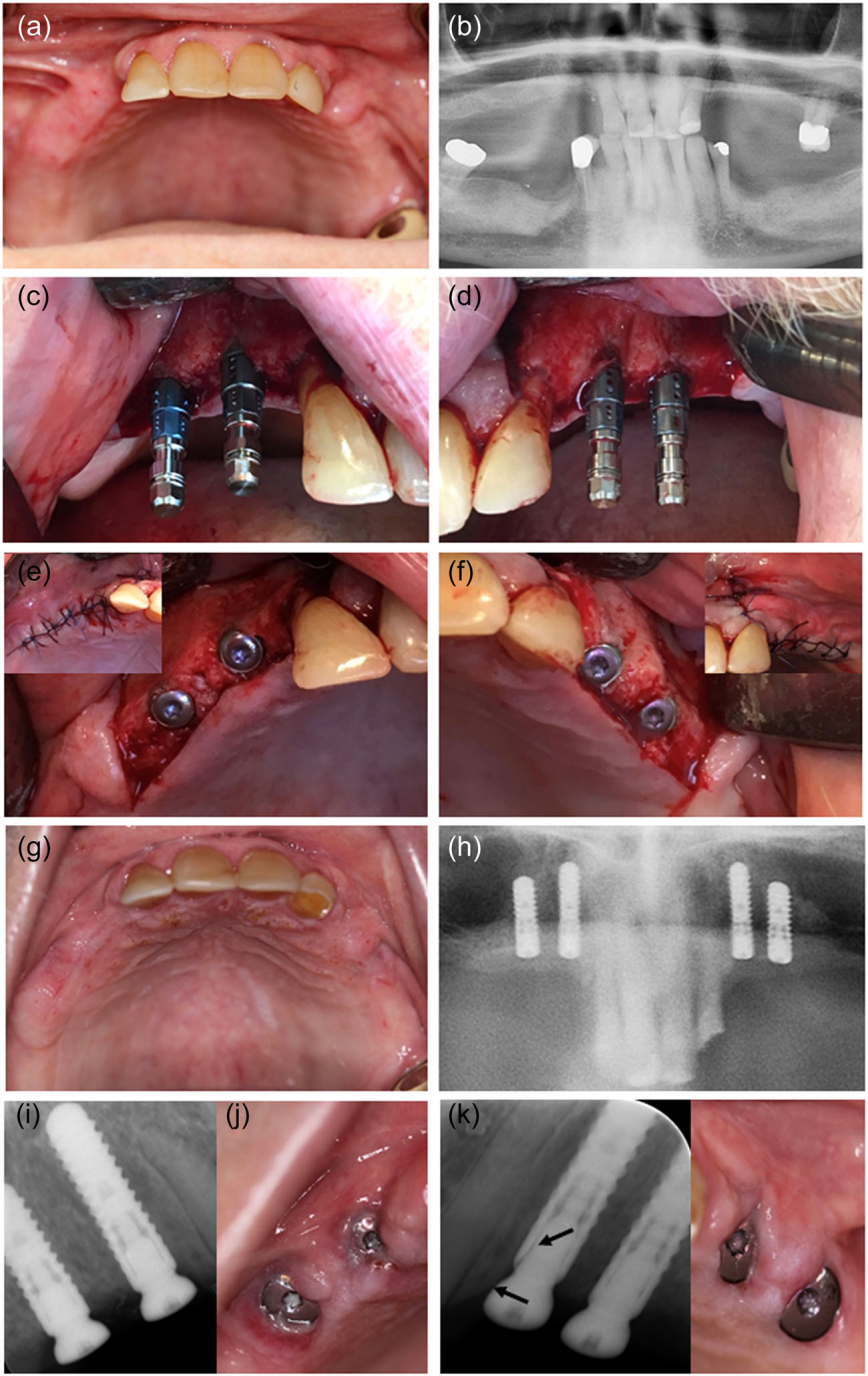
Implant surgery. (a, b) Before implant surgery, clinical photo, and panoramic radiograph. (c‐f) Implant surgery. (g, h) Healing before abutment surgery, clinically and on panoramic radiograph. No signs of bone degeneration around dental implants. (I, k) Periapical radiographs immediately after abutment surgery. The healing abutment in region 23 which was not completely in place (arrow) probably due to interference with the alveolar crestal bone distal to the lateral incisor (arrow). (j, l) Two weeks after abutment surgery, no signs of infection on the left side. Minor mucosal reaction distally around abutment at implant in the region 14.

Preoperative recordings were completed with the following results: Prostate cancer with metastases since 2015 treated with enzalutamid and denosumab for a duration of 13 months. He was a former smoker with no allergies but took different kinds of medicine due to a variety of comorbidities including hypertension and diabetes mellitus type 2 (Table 1). The clinical examination included dental registrations, periodontal probing, and a clinical evaluation of the signs of infection or necrotic bone. It revealed no pathological findings. Radiographically, he was assessed by panoramic X‐ray and cone beam computed tomography (CBCT). Based on the clinical and radiographic findings, four implants were planned in the regions 14, 13, 23, and 24 to support two cantilever FDPs. The implant operation was performed under local anesthesia and the standard protocol for staged implant placement was followed (Straumann Roxolid Bone Level; Straumann AG). The implants healed submerged for 3 months with an internal healing cap. Bone augmentation was not performed. Prophylactic antibiotics were administered 2 days preoperatively and continued for 9 days postoperatively with 500 mg amoxicillin and 125 mg clavulanic acid tablets (three times daily). The patient's upper RDP was re‐customized and reinserted 5 weeks after the implant surgery. Three months following implant insertion, the patient was recalled for clinical and radiographic follow‐up, where he also underwent an abutment operation under prophylactic antibiotics administrated as a one‐shot prophylaxis with 1000 mg amoxicillin and 250 mg clavulanic acid tablets. A high sound of percussion and no signs of infection were recorded at the follow‐up. Figure 1 summarizes the dental implant and abutment operations. A X‐ray was taken in both sites just after the abutment operation. Retrospectively, it was ascertain that the healing abutment in region 23 exerted pressure against the crestal bone mesially that is, at the oblique bone crest from region 22 to 23. Furthermore, it was observed that the healing abutment, as a consequence of the nonpassive fit/bone pressure, was pressed away from the mesial part of the fixture (Figure 1k).

**Table 1:** Patient characteristics

Time	Diagnoses	Medication	
At implant operation	Prostata cancer with bone metastases	Enzalutamid (Xtandi®)	
		Denosumab (Xgeva®)^a^	
	Hypertension	Acetylsalicylic acid	
		Atorvastatin	
		Furosemid	
		Kalium chloride	
		Lorsatan	
		Metoprolol	
	Diabetes mellitus	Metformin	
Postimplant operation	Prostata cancer with bone metastases	Cabazitaxel	
		Denosumab (Xgeva®)^a^	
		Abirateron	
		Prednisolone	
	Hypertension	Bendroflumethiazid, kalium	
		Atorvastatin	
		Calcium	
		Lorsatan	
	Atrial fibrillation	Edoxaban	
	Diabetes mellitus	Metformin	
	Periimplantitis, MRONJ	Amoxicillin, clavulanic acid	

Abbreviation: MRONJ, medication‐related osteonecrosis of the jaw.

^a^ Paused.

### 2.3 Implant‐supported FDPs

**Figure 2:**
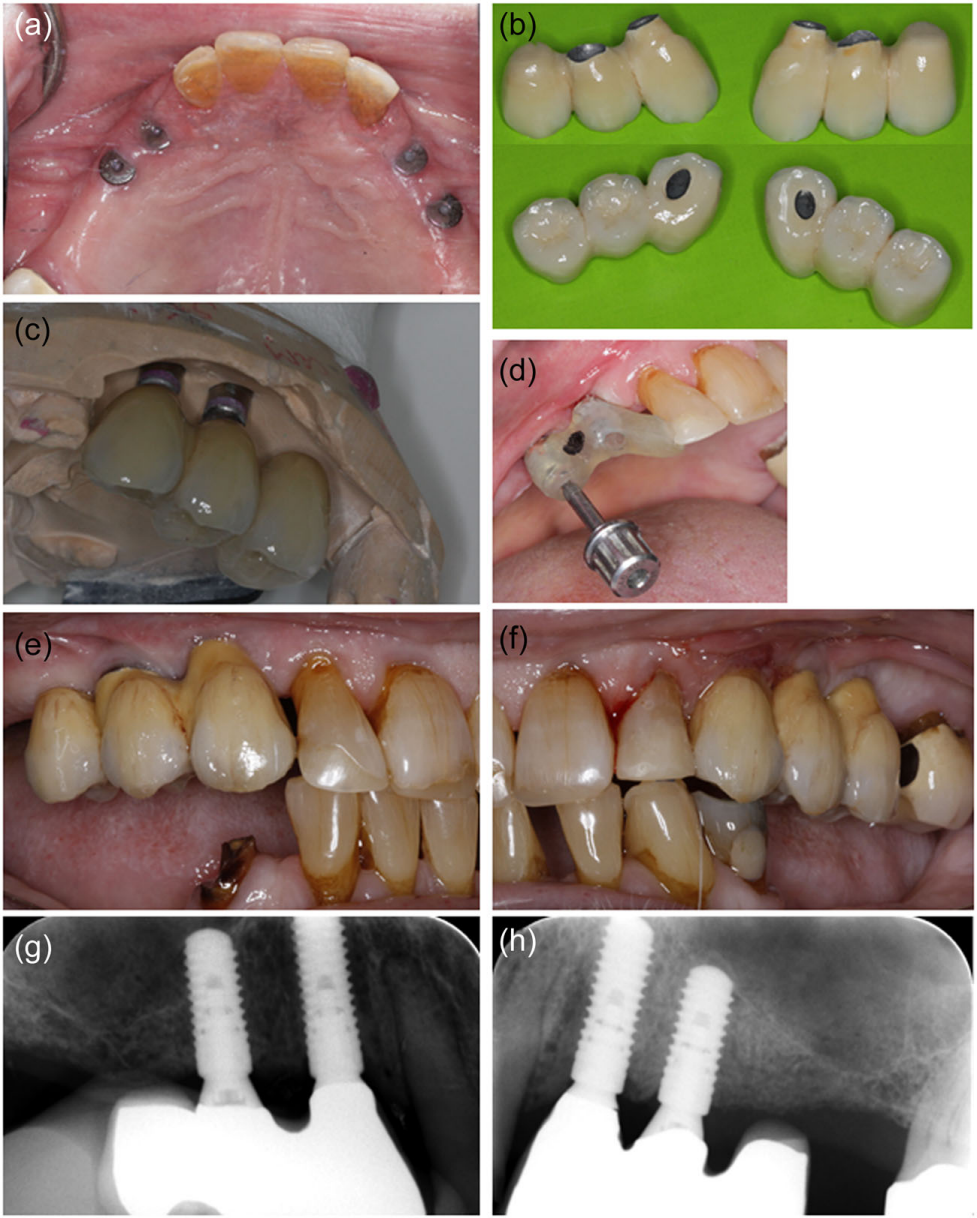
Prosthetic treatment. (a) Abutments at referral. (b) and (c) Implant‐supported restorations. (d) Installation using individual guide. (e‐g), and (h). Clinical and radiographic situation immediately after installation. Erythematous mucosa was observed in region 23. The minor bleeding in region 22 (f), is due to the newly performed composite restoration.

Three months after the abutment operation the prosthetic procedure was initiated at the Department of Oral Rehabilitation, University of Copenhagen. OHRQoL questionnaire OHIP‐49 gave a sum score of 35. The preliminary clinical examination showed a DMFT (decayed, missing, filled teeth) of 23, moderate visible amounts of plaque, and bleeding by probing, but no probing depth >4 mm at teeth or implants. Implant stability quotient (ISQ) was measured to 73−76 (Osstell ISQ Scale 0−100) and all implants had a high sound on percussion and no mobility. The minor signs of inflammation (tenderness/light pain and redness) around the healing abutments in regions 23 and 24 were initially interpreted as mucosal proliferation (Figure 2a). Impressions were taken using an open tray technique with polyether impression material. Impression pick‐ups from Straumann AG were used.

One month after impressions, the patient met for a try‐in of the metal skeleton of the implant‐supported constructions. The access for interproximal cleaning at the supraconstruction was controlled and sufficient. However, it was difficult to get the construction to fit, due to soreness of the mucosa, in regions 23 and 24. A preload was registered at the construction in the left side, and new occlusion registrations were taken. Three weeks later the patient had the implant‐supported constructions installed. Erythematous gingiva was documented around the implant in region 23 distally to the abutment (Figure 2a), probing depth was <3 mm and no suppuration was recorded around any of the reconstructions. Probing was painful for the patient, and he was tender to palpation apically to the implant. However, the constructions were installed. The screw‐retained cantilever FDPs were fabricated as metallic‐ceramic bridges using Straumann Variobase® abutments (13, 23) combined with Straumann Anatomic abutments (14, 24) in titanium. Figure 2 summarizes the prosthetic procedures.

### 2.4 Postimplant treatment

**Figure 3:**
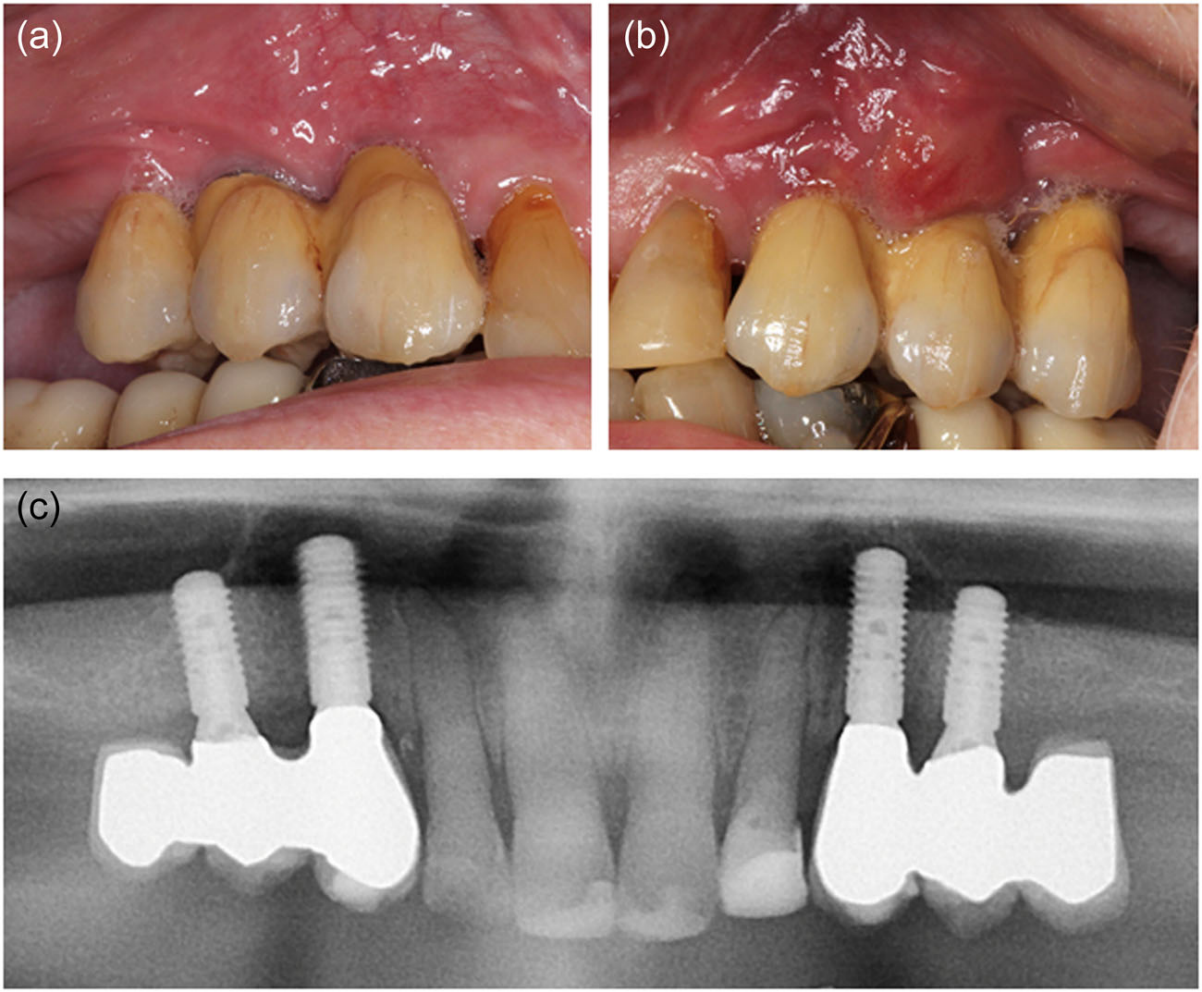
Treatment of minor peri‐implant abscess facially to implant 24. (a) Right side, no signs of any pathological conditions. (b) Minor abscess facially to dental implant 24. (c) Panoramic radiograph revealed no signs of bone degeneration around the dental implants.

Shortly after installation of the FDPs, the patient fractured a premolar in the mandible and contacted the Department of Oral & Maxillofacial Surgery. The patient was examined, and a surgical tooth extraction of the premolar was planned. In relation to the tooth extraction, the denosumab was discontinued. Due to persistence and aggravation of pain from the left side of the maxilla, the implants were examined too. A small abscess and probing depth of 5−8 mm with bleeding and suppuration were recorded on the facial aspect of the dental implant 24. A periapical radiograph did not reveal signs of peri‐implant bone loss in regions 23 or 24, Figure 3. Drainage of the abscess was instantly performed and the patient was started on antibiotic treatment for 7 days with 500 mg amoxicillin and 125 mg clavulanic acid tablets (three times daily). Peri‐implantitis 23, 24 was diagnosed. Surgical treatment of the peri‐implantitis was planned. No subjective or objective findings were recorded related to the implant treatment in the right side of the maxilla.

**Figure 4:**
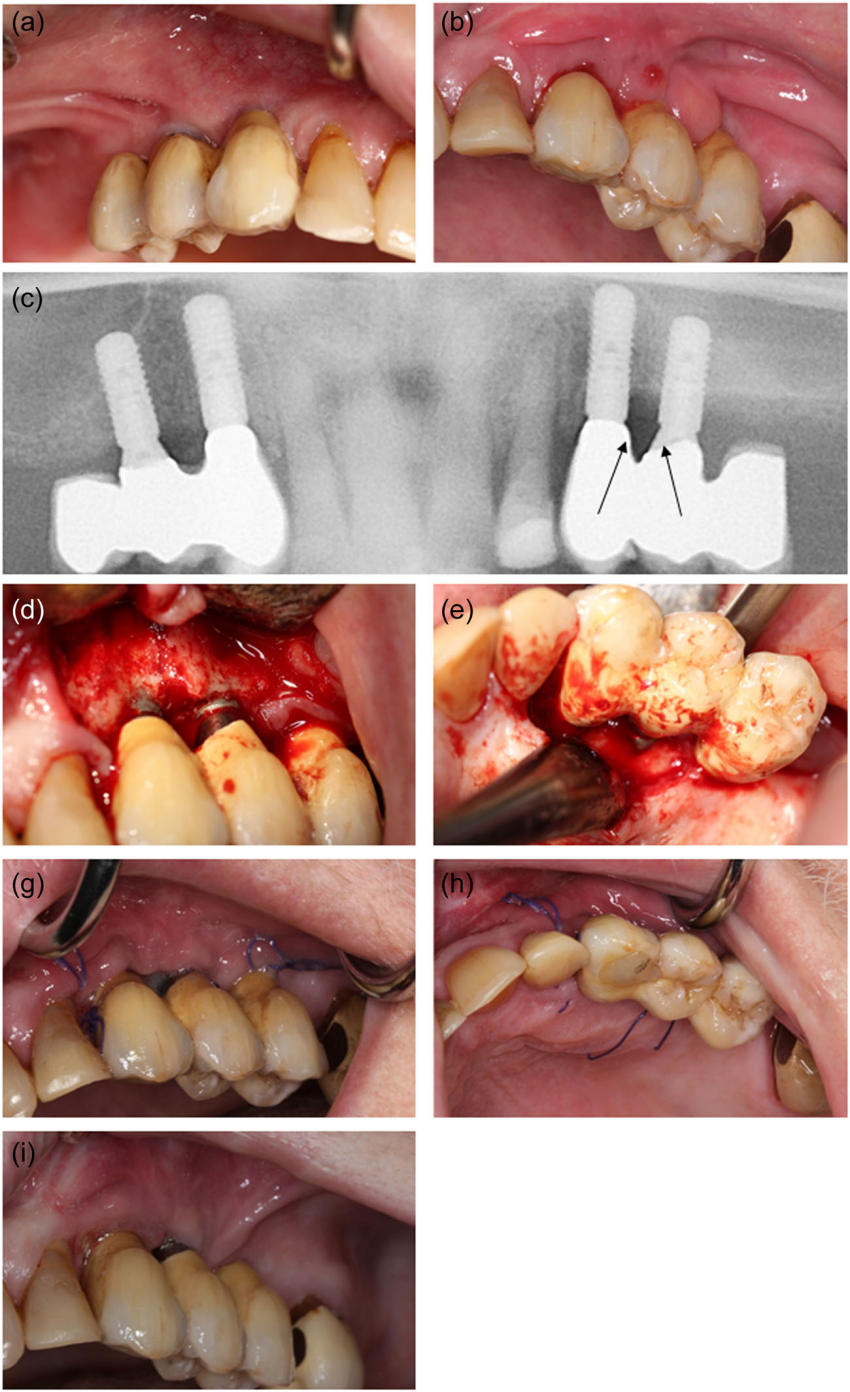
Peri‐implantitis treatment. (a) Right side, no signs of pathologic conditions. (b) Left side, edematous mucosa facially to dental implant 24. (c) Panoramic radiograph shows sign of bone degeneration approximal to implants 23 and 24 (arrows). (d, e) Periimplantitis operation, no signs of bone necrosis, picture taken after removal of granulation tissue. (g, h) Fourteen days postoperatively at suture‐removal. No signs of infection. (i) One month postoperative follow‐up. No sign of any pathologic conditions.

The operation was performed using local anesthesia (Lidocaine epinephrine 10 mg/ml, 5 microg/ml). The oral cavity was rinsed with 0.12% chlorhexidine preoperatively. The peri‐implantitis operation procedure included a crestal incision, combined with two facial releasing incisions, followed by raising a mucoperiosteal flap. Removal of substantial amounts of granulation tissue. The implant surfaces were mechanically debrided with titanium curettes and ultrasound equipment and cleaned with saline and 0.12% chlorhexidine. Then, the flap was repositioned, and the wound margins were reapproximated with single interrupted sutures, Ethicon Vicryl Suture 4‐0 (Johnson & Johnson). Antibiotics were administrated for 10 days postoperatively with 500 mg amoxicillin and 125 mg clavulanic acid tablets (three times daily). The sutures were removed 14 days following the surgery. Figure 4 summarizes the peri‐implantitis operation. At 1 month follow‐up, the patient had healed uneventfully.

**Figure 5:**
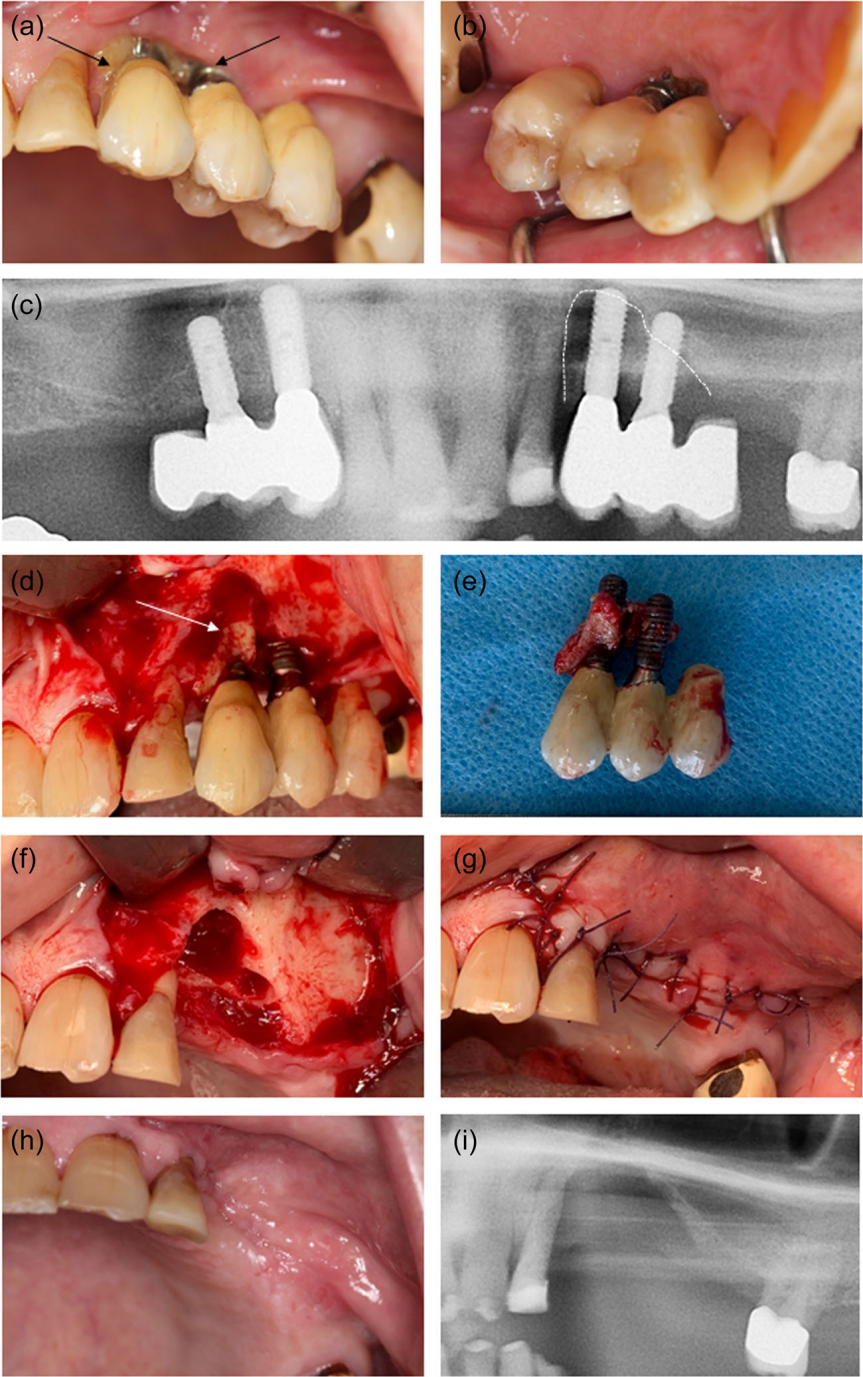
Removal of dental implants and resection of necrotic bone. (a, b) Six months follow‐up revealed necrotic bone (arrows) facially to implants 23 and 24. Patient had initially no pain or discomfort. (c) Panoramic radiograph indicating a sequestrum around dental implants 23 and 24 (dotted line). No pathologic conditions on right side of the maxilla. (d) Intraoperative, sequestrum facially to dental implant 23 (arrow). (e) Removal of dental implant restoration, with minor sequestrum attached to dental implant 23. (f) Before wound closure, clinically vital bone. (g) Tension‐free wound closure with interrupted single sutures, Vicryl 4‐0. (h,i) One‐month follow‐up clinical and radiographical. Uneventful mucosal healing.

At 6 monthly follow‐up at the Department of Oral Rehabilitation, the patient was informed of metastatic progression. The patient had started intravenous chemotherapy (Cabazitaxel) and anticoagulant medication (Lixana) due to atrial fibrillation. The patient did not report any discomfort or oral pain. The clinical examination showed no pathological findings from the implants in the right side of the maxilla (14, 13). The construction on the left side of the maxilla (23, 24) was mobile. Probing depths of 7−14 mm with bleeding and suppuration were recorded. The patient reported pain on probing and palpation in the region. A periapical radiograph showed extensive bone loss around the implants. OHIP‐49 sum score was 47. The patient was given an acute appointment at the Department of Oral and Maxillofacial, Copenhagen University Hospital the next day. The patient was assessed clinically again and had a panoramic X‐ray and a CBCT scan taken. Subsequently, the patient was diagnosed with MRONJ and resumed antibiotic treatment with 500 mg amoxicillin and 125 mg clavulanic acid tablets (three times daily). Pain from the jaw developed, and resection of the necrotic bone, including removal of the dental implants, was planned. The patient completed the MRONJ operation in general anesthesia 8.5 months after the installation of the implant‐supported constructions. The operation was performed under general anesthesia. Granulation tissue and necrotic bone were identified in regions 22, 23, and 24. The peri‐implantitis had further developed around 23. The implants were removed, and resection of necrotic bone was completed. Figure 5 summarizes the MRONJ resection and removal of the implants. Prophylactic antibiotics were administrated 2 days preoperatively and continued for 10 days postoperatively with 500 mg amoxicillin and 125 mg clavulanic acid tablets (three times daily). The patient was discharged the day after.

At the 1‐month follow‐up after MRONJ surgery the patient had healed uneventfully and received prosthetic treatment with a new removable maxillary RDP. The patient got his RDP 2 months after the surgery.

At the 1‐year follow‐up visit at the Department of Oral Rehabilitation, the patient was satisfied with the prosthetic solution and did not report any pain. Unfortunately, the cancer had progressed, and the oncologist could not offer any additional treatment. The clinical and radiographic examination showed no pathological findings related to the implant treatment in the right side of the maxilla (14, 13) and no signs of MRONJ. OHIP‐49 sum score was 2. The patient died 5 months later.

## 3 DISCUSSION

The present case report documents that dental implant treatment may be feasible in a patient on HDAR (denosumab); however, not without risks. Four implants were bilaterally inserted simultaneously in the maxilla, two in each side. The right side healed uneventfully and was successfully treated prosthetically, whereas MRONJ developed on the left side. Therefore, the primary goal of the patient—to avoid a removable prosthesis—was only accomplished for a short time, and the overall treatment was considered a failure.

There is a clear lack of literature regarding the risk of implant failure in patients with a history of high‐dose denosumab. However, based on the risk of developing MRONJ after other types of dentoalveolar surgery, dental implant treatment is still by many considered contraindicated in these patients (Stavropoulos et al., 2018). In a systematic review from 2021 evaluating the safety of placing implants in patients with a history of antiresorptive drug therapy, only one case series study reported on denosumab therapy and dental implants in osteoporotic women (Sher et al., 2021). The authors concluded that osteoporotic patients treated with denosumab have a negligible risk of developing MRONJ after implant placement, and that “there is a lack of data available in the literature regarding the risk of developing MRONJ after implant placement in cancer patients treated with denosumab” (Sher et al., 2021).

It has previously been documented that primary healing can be accomplished after surgical tooth extractions in patients with cancer on HDAR applying low‐trauma surgery including primary wound closure (Ottesen et al., 2022). It was therefore hypothesized that dental implant treatment could be feasible by applying the same surgical principles, and promising early results have been reported (Andersen et al., 2022). The patient in this report presented with a number of MRONJ risk factors: first of all, he was in HDAR, and additional antiangiogenetic and immunosuppressive medications may contribute to an even higher risk of MRONJ development. Additionally, diabetes as well as the intake of corticosteroids and chemotherapy affect the immune system and may increase the susceptibility to infection (Otto et al., 2021). Despite massive research efforts to identify the pathogenesis in relation to MRONJ, it is still not fully understood. However, local infection has been proposed as a possible hypothesis (Nicolatou‐Galitis et al., 2019; Otto et al., 2021), and Pichardo et al. suggest that MRONJ may not develop spontaneously without the presence of local infection, for example, peri‐implantitis (Pichardo et al., 2020).

Interestingly, uneventful healing was observed bilaterally after implant placement. No signs of inflammation were present at the second stage of surgery and osseointegration was achieved as evaluated clinically. However, clinical photos and radiographs from the prosthetic startup 3 months after the abutment operation showed minor mucositis around the healing abutment in the regions 23 and 24 (Figure 2a), where it appears from the radiograph (Figure 1k) that the healing abutment in region 23 was not completely in place probably due to interference with the alveolar crestal bone distal to the lateral incisor. This oblique alveolar bone crest anatomy in an area with dentition facing an edentulous area requires the use of a narrow and long healing abutment, which does not interfere with the biological distance to the marginal bone. If a short, standard abutment with an excessively wide neck is used, as in the present case, it is almost impossible to place the healing abutment in the fixture without inducing high stresses in the bone. Such stress concentrations in the bone normally induce minor inflammatory reactions activating and stimulating the lining cells and osteoblast to produce osteoid and more mineralized bone adjacent to the implants (Gotfredsen et al., 2002). In systemically healthy patients, such situations usually result in crestal bone remodeling and eventually a loosening of the healing abutment, when the overloaded crestal bone is resorbed. The patient did not present a loose abutment in region 23. After bone remodeling and tightening of the healing abutment, a new steady state will follow, and the prosthetic procedures can be performed with a reestablished biological width. Such spontaneous healing with bone remodeling may not be feasible in a patient such as the present case with compromised healing potential. Instead, unprogrammed cell death caused by cell injury may have occurred, resulting in necrosis of the bone (Aguirre et al., 2021). Initially, the patient, in this case, had an abscess from the approximal region 23 and 24, and a recurrent fistula in the same region. The radiographs showed bone loss approximal between 23 and 24, not mesial to the implant 23 where it would have been anticipated from the misfit of the healing abutment. The same biological events may be hypothesized to follow a misfit of the implant‐supported construction resulting in a preload of the alveolar bone. Thus, the fact that the prosthetic reconstruction was tried‐in several times, could also have induced inflammation or triggered an existing inflammation in the area (Fickl et al., 2021). The prosthetic reconstruction not using standard screw‐retained bridge abutment but instead combining a screw‐retained, Variobase® abutment in the canine region with a customized prepared anatomic abutment in the premolar region is technically challenging and the risk for misfit and preloads are increased. Retrospectively, these challenging constructions were not favorable in such a compromised patient, and simple solutions should have been preferred to decrease the risk of inflammatory reactions in the alveolar bone.

Another potential eliciting factor for the unsuccessful outcome may be the sudden change in medication as the patient started new chemotherapy 4 months after insertion of the FDPs.

Re‐evaluating the patient's clinical photos and radiographs from the peri‐implantitis operation, the peri‐implant bone defect did not have the classical bowl shape of peri‐implantitis, but rather a vertical crestal bone resorption (Figure 4d). It is reasonable to believe that the inflammatory reactions initially diagnosed as peri‐implantitis instead were the initial stages of MRONJ. In this case, the MRONJ developed in the maxilla. Only one‐third of MRONJ cases affect the maxilla, but with a predilection to the premolar region as in this patient (Otto et al., 2021).

During the implant treatment, the patient completed the OHIP‐49 questionnaire, which consists of 49 statements and has a sum score from 0 to 196, where a high score represents a low OHLQoL (Gjørup & Svensson, 2006; Slade & Spencer, 1994). This patient had a lower sum score at baseline examination just after placing the FDPs than at the 6‐month follow‐up. This follow‐up was also the time for the peri‐implantitis diagnosis, where the patient had pain. After removal of the implants in the left side of the maxilla, the sum score decreased as an indicator of increased OHLQoL.

It is interesting that this patient had two identical treatments in two identical regions of the maxilla but only one side failed, whereas the contralateral was without any complications. This indicated that the prosthetic preload or other trauma in combination with heavy medication especially HDAR, chemotherapy, and immunosuppressive medication may result in MRONJ. However, it must be recognized that successful implant treatment has been documented using the same surgical and prosthetic protocol in patients with cancer on similar polypharmaceutical medication without development of MRONJ (Andersen et al., 2022), which emphasizes that the present case was serious and rare.

## 4 CONCLUSION

HDAR should still be considered a risk factor for MRONJ development in dental implant treatment, especially in presence of comorbidities (e.g., diabetes mellitus) and polypharmacy (e.g., chemotherapy and steroids). In such patients, minimal trauma during surgery and prosthodontics is crucial to increase the chance for successful healing.

## AUTHOR CONTRIBUTIONS

All authors designed this case report. Camilla Ottesen wrote the first manuscript draft. Camilla Ottesen and Sanne W. M. Andersen designed the figures. Sanne W. M. Andersen, Klaus Gotfredsen, Simon S. Jensen, and Thomas Kofod supervised and provided their expertize in the manuscript writing. All authors approved the article.

## CONFLICT OF INTEREST

The authors declare no conflict of interest.

## ETHICS STATEMENT

Written informed consent was obtained from the patient for participation in and publication of the dental implant treatment. No personal details of the patient have been included in the manuscript. Approval to perform the study in which this patient was mistakenly enrolled was provided by the Danish National Committee on Health Research Ethics (No. H‐17025531) and by the Danish Data Protection Agency (No. 2012‐41‐0045). The study followed the Declaration of Helsinki (2013) and is registered in clinicaltrials.gov (NO. NCT04741906).

## Data Availability

Data are available from the authors on reasonable request.
